# Carbon nanotubes promote cell migration in hydrogels

**DOI:** 10.1038/s41598-020-59463-9

**Published:** 2020-02-13

**Authors:** Hossein Ravanbakhsh, Guangyu Bao, Luc Mongeau

**Affiliations:** Department of Mechanical Engineering, McGill University, Montreal, QC H3A0C3 Canada

**Keywords:** Biomaterials - cells, Biomedical engineering

## Abstract

Injectable hydrogels are increasingly used for *in situ* tissue regeneration and wound healing. Ideally, an injectable implant should promote the recruitment of cells from the surrounding native tissue and allow cells to migrate freely as they generate a new extracellular matrix network. Nanocomposite hydrogels such as carbon nanotube (CNT)-loaded hydrogels have been hypothesized to promote cell recruitment and cell migration relative to unloaded ones. To investigate this, CNT-glycol chitosan hydrogels were synthesized and studied. Chemoattractant-induced cell migration was studied using a modified Boyden Chamber experiment. Migrated cells were counted using flow cytometry. Cell adhesion was inferred from the morphology of the cells via an image segmentation method. Cell migration and recruitment results confirmed that small concentrations of CNT significantly increase cell migration in hydrogels, thereby accelerating tissue regeneration and wound healing in situations where there is insufficient migration in the unloaded matrix.

## Introduction

Hydrogels offer many advantages for biomedical applications due to their unique properties, in particular, their biocompatibility, biodegradation rate, mechanical stiffness, hydrophilicity, and porosity^1,2^. Nanocomposite hydrogels have the potential to further improve upon these properties through the addition of nanoparticles to pure hydrogels. Such nanocomposite hydrogels have been reported to increase three- and four-dimensional printability, stiffness tunability, and electrical conductivity^3–9^. For tissue engineering applications, composite hydrogels should allow sufficient cell adhesion and cell migration within its network. Such cell-biomaterial interaction is known to accelerate wound healing and tissue regeneration in the target region^10,11^. Collagen, fibrin, fibronectin, nanoclays, cellulose nanocrystals, cellulose nanowhiskers, graphene oxide, and carbon nanotubes (CNTs) are among the many functional reinforcements available for fabricating bionanocomposite hydrogels^7,12–16^. In our previous work, we found that the addition of CNTs to a glycol-chitosan matrix alters gelation time, porosity, and storage modulus^17^.

Since their discovery in 1991 by Iijima^18^, CNTs have been broadly used in biomedical applications. The CNTs can firmly attach to proteins and receptors on the cell membrane. The adherence between CNTs and cells is important. Firstly, single-walled carbon nanotubes (SWCNTs) may be employed for the purpose of drug delivery^19^. Gangrade *et al*. took advantage of the thermal properties of the SWCNTs to obtain a temperature-dependent release of preloaded doxorubicin^20^. Secondly, multiwalled carbon nanotubes (MWCNTs), which due to their size are more practical for tissue engineering applications, can be used as focal adhesion sites to enhance cell adhesion for anchorage-dependent cells such as fibroblasts^21,22^. The latter may lead to an increased cell migration rate in CNT-loaded composite hydrogels. The larger diameter of MWCNTs with respect to SWCNTs approximates the physical characteristics of fibrous proteins, such as collagen and elastin, in the extracellular matrix (ECM). This idea is feasible based on recent findings. It was reported that CNTs offer focal adhesion sites for cells, and promote migration through the hydrogel matrix^22–25^.

Scratch assays, transwell assay systems, cell-exclusion zone assays, microfluidic-based assays, and radial cell migration protocols have been used to characterize cell migration in two-dimensional (2D) cell culture environments^26–30^. Most of these methods are focused on cells’ behavior rather than on cell-material interactions. Fewer methods are available for three-dimensional (3D) environments, whereby migration is usually quantified based on the distance traveled by the cells or the number of migrated cells^31^. Li *et al*. used human hepatocyte growth factors and cell culture inserts to demonstrate the effects of growth factor-loaded nanoparticles on cell migration in hydrogels^32^. They used a confocal microscope to count the number of migrated cells in random zones in the hydrogel. Heris *et al*. also used confocal microscopy, but in time-lapse, to evaluate the average speed of cells that were homogenously encapsulated in hyaluronic acid/gelatin composite hydrogels^11^. In the absence of any directional constraints, however, the random motion of the cells tends to dominate motility statistics, which may be misleading. A method that leads cells across the scaffold boundary would help better characterize the ability of a priori acellular materials to recruit cells from their surrounding environment. The optimum design for cell migration experiment is, therefore, a configuration that can capture the total number of migrated cells and minimize the influence of the cells’ random motion or possible biases due to hydrogel swelling over the duration of the experiment.

In general, cell migration and cell adhesion are essential to the wound healing process^33,34^ and tissue regeneration. By studying the impacts of adding covalently grafted adhesion ligands and epidermal growth factor to polyethylene glycol hydrogels, Gobin and West demonstrated the importance of cell adhesion in promoting cell migration in hydrogels^35^. Latifi *et al*. qualitatively showed that adding collagen type III to glycol chitosan (GC) hydrogel increases the fibroblasts attachment to the surface of the hydrogel^36^. To quantify cell adhesion, previous researchers have used cell morphology such as area, axial ratio, perimeter, major axis, and minor axis^37,38^. The mechanical viscoelastic properties of the hydrogel are also an important factor for adhesion. Van den Broeck *et al*. successfully modulated the mechanical properties of CNT/polyethylene glycol composite hydrogels by changing the concentration of MWCNTs, without jeopardizing the cytocompatibility of the biomaterial^24^. The modulation of the stress relaxation time of the composite hydrogels can affect their performance in terms of cell adhesion^39^.

It has been previously reported that the mechanical properties of the GC hydrogel such as the storage and loss modulus are similar to those of human soft tissues. Here, CNTs were added to a GC hydrogel matrix to find the influence of CNT concentration on cell recruitment, cell migration, and cell adhesion. A radial cell recruitment assay and a modified Boyden Chamber experiment, along with flow cytometry, were employed to study cell migration in CNT/GC hydrogels. Cell adhesion was quantified using a novel image segmentation method. A new parameter, the extensibility ratio, was introduced and used to compare cell adhesion in different samples. The biodegradation of the hydrogels with different CNT concentrations was assessed over time. The stress relaxation of CNT composite hydrogels was also characterized to investigate its effect on cell migration. Since our previous cell viability experiments were conducted with immortalized human vocal fold fibroblasts^17^, we use the same cell line encapsulated in composite CNT/GC hydrogels to perform the biological experiments. The same protocols are broadly applicable to any cell-material interactions.

## Materials and Methods

### Material preparation

Multiwalled carboxylic (COOH) functionalized CNTs (US Research Nanomaterials, Inc.) with an outer diameter of 50–80 *nm* were used to prepare fibrous composite hydrogels. We used surfactants to facilitate CNT dispersion. Triton X-100 (Acros Organics) with concentrations of 10%, 1%, and 0.25% (*vol*/*vol*) was used to identify the minimum surfactant concentration needed to break up CNT clusters. The CNT stock solution with a concentration of 2000 *μg*/*ml* was sonicated using a bath sonicator (Branson Ultrasonics, Danbury, CT) for 5 minutes prior to hydrogel preparation. Bath sonicators deliver less acoustic power to the nanotubes than probe sonicators. Moderate sonication enhances homogeneity with little damage to the CNTs.

Glycol chitosan powder (Chemos GmbH, Germany) was dissolved in 1x phosphate-buffered saline (Pbs) with a concentration of 5% using a tube rotator (Fisher Scientific) at 25 *rpm* for 24 hours. The hydrogels were synthesized such that final concentrations of 2% for glycol-chitosan, 0.005% for glyoxal (Sigma-Aldrich Corporate) as the crosslinker, and various concentrations of COOH-CNTs were achieved. Hydrogels made of CNT/GC with concentrations of 0,250,500,750 *μg*/*ml* (denoted as Control, CNT250, CNT500, CNT750, respectively) were defined as the study groups. All materials were sterilized in an autoclave (Tuttnauer™, Model #2540) prior to hydrogel preparation. The biological experiments were conducted in a cell culture hood.

### *In vitro* cell culture

The fibroblasts were cultured in Dulbecco’s Modified Eagle Medium (DMEM) supplemented with 10% fetal bovine serum (FBS), 1% non-essential amino acids, and 1% penicillin/Streptomycin (Sigma-Aldrich Corporate). The cells were incubated at 37° C in a humidified incubator (NuAire DHD AutoFlow, Model #5510) with 5% *CO*_2_. The cell culture medium was replaced with fresh medium every 3 days. The cells were passed every week at a confluency of about 80%. A Bright-Line hemocytometer (Sigma-Aldrich Corporate) was used to roughly count the number of cultured cells before encapsulation in the hydrogels.

### Cell recruitment

Hydrogel samples were prepared in plastic vials, as described before. Prior to gelation, 50 *μl* of the cell-free hydrogel was placed around the center point of each well in an 8 well *μ*-slide (Ibidi, Martinsried, Germany) to form a semispherical shape. The samples were then incubated for 45 mins until they were fully crosslinked. The cells were passed and stained using Vybrant® Dil cell-labeling solution (Invitrogen, Carlsbad, CA). A volume of 200 *μl* of the cell solution, with a concentration of 500,000 cells per *ml*, was added to each well to cover the hydrogel droplet. To ensure the stability of the cells and the hydrogel, the samples were incubated for 24 hours prior to imaging. A confocal laser scanning microscope (Zeiss LSM710, Oberkochen, Germany) was used to record cells’ movement over a period of 14 hours. The acquisition and analysis of the time-lapse images were conducted using Zen 2.6 (Zeiss, Oberkochen, Germany) and Imaris 8.3.1 (Bitplane, Zurich, Switzerland), respectively. The regions of interest for imaging were the border edges of hydrogel droplets, where the cells may penetrate the hydrogel.

### Chemoattractant-induced cell migration

Cell migration in composite hydrogels with different COOH-CNT concentrations was studied using a modified Boyden Chamber experiment. A gradient of chemoattractant media was employed to initiate cell mobility. Delta-treated polycarbonate cell culture inserts with a pore size of 8 *μm* in 12-well plates (Thermo Scientific Nunc™) were used for three-dimensional cell culture. The fibroblasts were passed, counted, and encapsulated in vials of hydrogels. Since the overall migration rate was predicted to be small^40^, we aimed for an initial population of 1~1.4 million fibroblasts in each insert to obtain enough migrated cells for comparison. Prior to gelation, 200 *μl* of the hydrogel was transferred from the vials to porous inserts. The samples were then incubated at 37 °C with 5% *CO*_2_ for one hour to complete the gelation process and expose the cells to a serum starvation period, which may accelerate migration. Afterwards, 200 *μl* of serum-free DMEM was added on the hydrogels’ surface, and the inserts were suspended over the 12-well companion plate, which contained 400 *μl* of completed DMEM + 10%FBS. Triplicates were used for each of the four study groups (Control, CNT250, CNT500, CNT750).

The samples were incubated for one week. The completed DMEM + 10%FBS was regularly replaced with fresh media every two days to ensure the stability of the chemoattractant gradient. On day 7, the media was removed, and the inserts were gently washed with PBS 1x (Wisent Inc., QC, Canada). The migrated cells were then dissociated from the bottom of the inserts by adding 200 *μl* of Trypsin-EDTA (0.25%*w*/*w*) and rocking the plate. After 2 minutes of incubation, 800 *μl* of fresh completed DMEM was added to the cell solution to avoid cell digestion. The migrated cells solution was then collected for cell counting with a flow cytometer.

### Flow cytometry

A FACSCanto^™^II flow cytometer (BD Biosciences, San Jose, CA) was employed to count the number of migrated cells. A volume of 50 *μl* of counting beads (Precision Count Beads^™^, Biolegend, San Diego, CA) with a concentration of 1.03 × 10^6^ *particles*/*ml* was added to each 500 *μl* of the cell solution prior to flow cytometry. The size and complexity of the beads are different from those of the fibroblasts. No staining was therefore needed to distinguish the beads from the cells. They were separated based on their forward scattered area (FSC-A) and side scattered area (SSC-A) signals. Flow cytometry-based cell counting highly depends on the precision of solution volumes. A reverse pipetting method was used when transferring solutions to minimize errors.

The FACSDIVA^™^ software, version 8 (BD Biosciences, San Jose, CA) was used for data acquisition. For each series, a volume of 550 *μl* of the cell solution was vortexed and loaded on the flow cytometer. The data was collected until 10^4^ events were recorded, or the sample was fully used, whichever happened first. Data analysis was conducted using the FlowJo^™^ software, version 10.5.3 (BD Biosciences, San Jose, CA). The number of counting beads and the number of migrated cells were determined by defining proper gates in FlowJo^™^, as discussed in section 3.3.

### Cell adhesion

The hydrogels with encapsulated fibroblasts were prepared as mentioned in sections 2.1 and 2.2. The cell density in the hydrogel was 4 × 10^5^ cells per *ml*. A volume of 200 *μl* of hydrogel was added in each of the 8 wells of a *μ*-slide, and the samples were incubated over one hour. A volume of 250 *μl* of cell culture media containing completed DMEM + 10% FBS was then added to the samples. The hydrogel samples were incubated for 72 hours to allow enough time for the cells to adhere to the hydrogel network. On day 3, the cells were fixed using 3.7% methanol-free formaldehyde (Acros Organics) and permeabilized with Triton X-100. The samples were immersed in 1% bovine serum albumin (Sigma-Aldrich Corporate) solution for 30 minutes to decrease possible background noise during imaging. Alexa Fluor^TM^ 633 Phalloidin staining solution (Invitrogen, Carlsbad, CA, United States) was used to stain the F-actin filament of the fibroblasts. The cells’ nuclei were also stained using DAPI. The samples were washed with prewarmed PBS, between each step. The samples were then imaged using the confocal laser scanning microscope. Subsequently, the images were processed in MATLAB^®^ to quantify the extent of cell adhesion based on the morphology of the F-actin filaments.

### Biodegradation

To examine the enzymatic biodegradation of the study groups, 1*ml* of hydrogel samples with the prementioned CNT concentrations were synthesized in 2*ml* Eppendorf vials. Using triplicates, the biodegradation behavior of the four study groups was examined on days 0, 1, 3, 7, 14, and 30, for a total of 72 samples. After complete gelation at 37 °C, all samples were frozen in −80 °C, and then lyophilized in a ModulyoD 5 L freeze dryer (Thermo Fisher Scientific) at 290 ± 10 *μbar* and room temperature. Both freezing and lyophilizing lasted 24 hours. A high-precision balance (Quintix, Sartorius, precision = 1*mg*) was then used to measure the dry weight of the hydrogels, *W*_*i*_.

The enzyme solution was prepared by dissolving lysozyme powder (Thermo Fisher Scientific, Approx. 20,000 units/mg dry weight) in PBS 1x. The concentration of lysozyme was 500 *μg*/*ml*, which is the same as that of the human airway and nasal fluid^41^. A volume of 1*ml* of enzyme solution was added to the freeze-dried hydrogels, and the samples were agitated using an orbital shaker (VWR 3500, Radnor, PA, United States) at 75 *rpm*. The enzyme solution was refreshed every other day to stabilize the enzymatic reaction. At each time step, the supernatant was removed from one set of samples, and stored in −80 °C. On day 31, when all the samples were frozen, they were lyophilized for a second time for 24 hours. The final dry weight of the samples, *W*_*f*_, was measured using the same balance. *W*_*i*_ and *W*_*f*_ are the hydrogels’ net weights, excluding the vial’s weight. The degradation of the samples was quantified as the percentage of remaining weight, %*RW*, i.e.

1 $$ \% RW=\frac{Wi-{W}_{f}}{{W}_{i}}\times 100$$

### Stress relaxation

A DHR-2 torsional rheometer (TA Instruments) with parallel plates was employed to measure the stress relaxation time. A volume of 350 µl of hydrogel was prepared and added directly on the bottom plate of the rheometer. The top plate was then lowered until the hydrogel completely filled the gap with a barrel-like shape. The gap was measured to be within the range of 800~1000 *μm*. The temperature was increased to 37 °C to resemble body temperature. The soaking time was 14 hours to ensure complete gelation. The samples were isolated with mineral oil and a solvent trap in order to minimize dehydration rate during soaking. A constant torsional strain of 10% was then applied to the hydrogel sample, and the corresponding stress in the hydrogel was recorded over a period of 3 hours. The recorded stress time history was used to quantify the stress relaxation rate for hydrogels with different CNT concentrations.

### Statistical analysis

The samples were triplicated for cell migration, stress relaxation, and biodegradation experiments. The built-in two-tailed student’s T-test function of MS Excel (Microsoft) was employed for analysis, using a *P-value* threshold of 0.05.

## Results

### CNT dispersion

Three different concentrations of Triton X-100, 10%,1%, and 0.25% (*w*/*w*), were prepared in deionized water to find the minimum concentration that leads to a stable well-dispersed COOH-CNT suspension. Carboxylic CNTs were dispersed in all three solutions with a concentration of 2 *mg*/*ml* using the protocol in section 2.1. Two hours after dispersion, the CNTs in 0.25% Triton X-100 solution agglomerated at the bottom of the glass tube. The two higher-concentration solutions remained stable for several weeks. A concentration of 1% Triton X-100 was therefore found to be functional for dispersing COOH-CNTs.

### Cell recruitment

**Figure 1:**
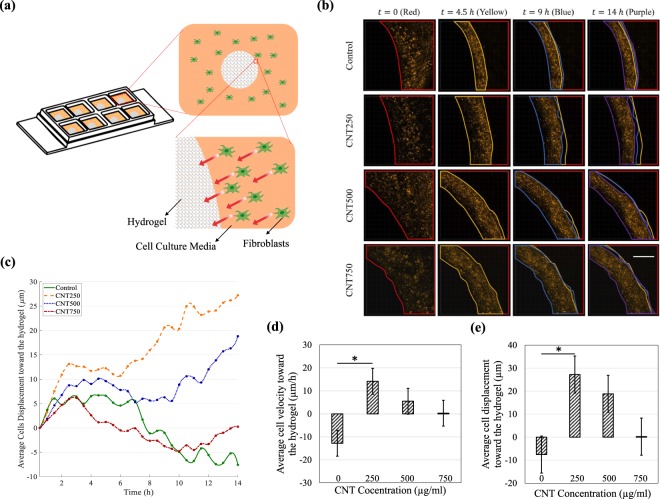
(**a**) Schematic configuration of the cell recruitment experiment. Due to their anchorage-dependence, the fibroblasts move toward the hydrogel droplet and in some cases penetrate it. (**b**) Time-lapse images of the cells (stained in orange) surrounding one drop of hydrogel (left side of the mosaic images). The regions that are occupied by the cells are illustrated in color-coded frames: red, yellow, blue, and purple, chronologically. The animated cell recruitment is shown in Supplementary Videos S1 to S4. The scale bar is the same for all the images and is equal to 400 *μm*. (**c**) The movement of the cells over time in terms of directional displacement. A positive displacement means that the cells are moving toward the center of the hydrogel, and vice versa. (**d**) fibroblasts’ average directional velocity at *t* = 14 *h*. (**e**) Fibroblasts’ average directional displacement at *t* = 14 *h*. The cells surrounding CNT250 hydrogel were recruited more effectively. *p < 0.05 corresponds to a significant difference.

As schematically shown in Fig. 1a, a droplet of the hydrogel is placed in an uncoated eight-well and surrounded by the fibroblasts. Due to the cells’ affinity to migrate toward regions with lower local cell-density^42^, they move toward the hydrogel droplet, which has no cells in it. The cells can attach to the hydrogels’ surface and in some cases penetrate it. The Supplementary Material (Videos S1 to S4) and the color-coded regions in Fig. 1b show how the cells move towards the hydrogel. The average displacement of the fibroblasts over time, plotted in Fig. 1c, shows that the CNT-loaded GC hydrogels generally recruit the fibroblasts better than the pure GC hydrogel, i.e. the control sample. The average velocity and displacement of the cells toward the center of the hydrogel droplet was measured at *t* = 14 *h* and plotted in Fig. 1d,e, respectively. The velocity and displacement of the cells are larger for the CNT250 hydrogel, thereby indicating more effective cell recruitment. For the control samples, both the velocity and the displacement have negative values, which shows that the hydrogel’s pore size is not large enough for the cells to penetrate the hydrogel. The fibroblasts moved toward the CNT-free hydrogel until they reached its surface. Afterwards, hydrogel swelling biased cell motion and made them move backwards with a negative velocity and displacement. Other drawbacks of this design are that the hydrogel droplet geometry was not reproducible, and hydrogels swelling over the imaging period was not considered. These flaws prompted us to find a better method to accurately quantify cell kinematics.

### Cell migration

**Figure 2:**
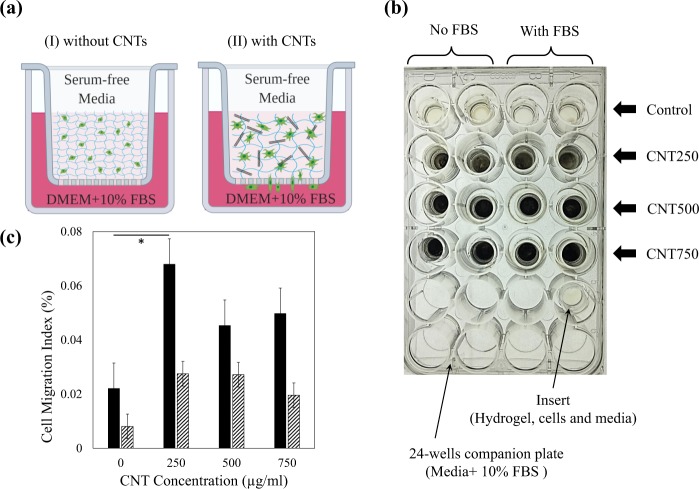
(**a**) Schematic of chemoattractant-induced cell migration assay using a modified Boyden Chamber experiment. Adding CNTs increased the hydrogel porosity and cell migration. (**b**) Samples prepared in porous cell culture inserts and 24-well plate. To check the effect of FBS, one-half of the samples, denoted as No FBS, had no chemoattractant. (**c**) The average cell migration index for the samples with (solid pattern) and without (diagonal pattern) chemoattractant. *p < 0.05 corresponds to a significant difference.

The configuration of the chemoattractant-induced cell migration and the effect of CNTs are schematically shown in Fig. 2a. The hydrogel was covered by serum-free media to avoid dehydration during the assay. The 10% FBS solution beneath the porous membrane served as the chemoattractant. The chemoattractant gradient directed fibroblasts motion across the porous polycarbonate membrane. For all hydrogels, two samples were prepared by replacing DMEM + 10% FBS with serum-free media to study the effect of chemoattractant. Two other samples were also studied using serum-free media in the bottom and DMEM + 10% FBS in the top of the cell-encapsulated hydrogel. The samples were prepared simultaneously in a 24-well companion plate, as shown in Fig. 2b. The cell migration index was defined as the ratio of the number of cells migrated through the porous membrane and the total number of encapsulated cells. As a preliminary study, the migrated cells solution was observed under a brightfield microscope (Olympus CKX41) to confirm that the cells migrated in the CNT/GC hydrogels. Flow cytometry was utilized to accurately count the number of migrated cells, as described in the next paragraph. The cell migration index obtained from flow cytometry is shown in Fig. 2c. The highest rate of cell migration was for the CNT250 group. A higher concentration of CNT did not necessarily increase cell migration further. As expected, the cell migration index for samples without FBS is relatively smaller but follows the same trend with respect to CNT concentration. The chemoattractant act as an accelerator for cell migration. No cell migration was observed for the samples with switched configuration of serum-free media and DMEM + 10% FBS. This again highlights the role of the chemoattractant agent.

**Figure 3:**
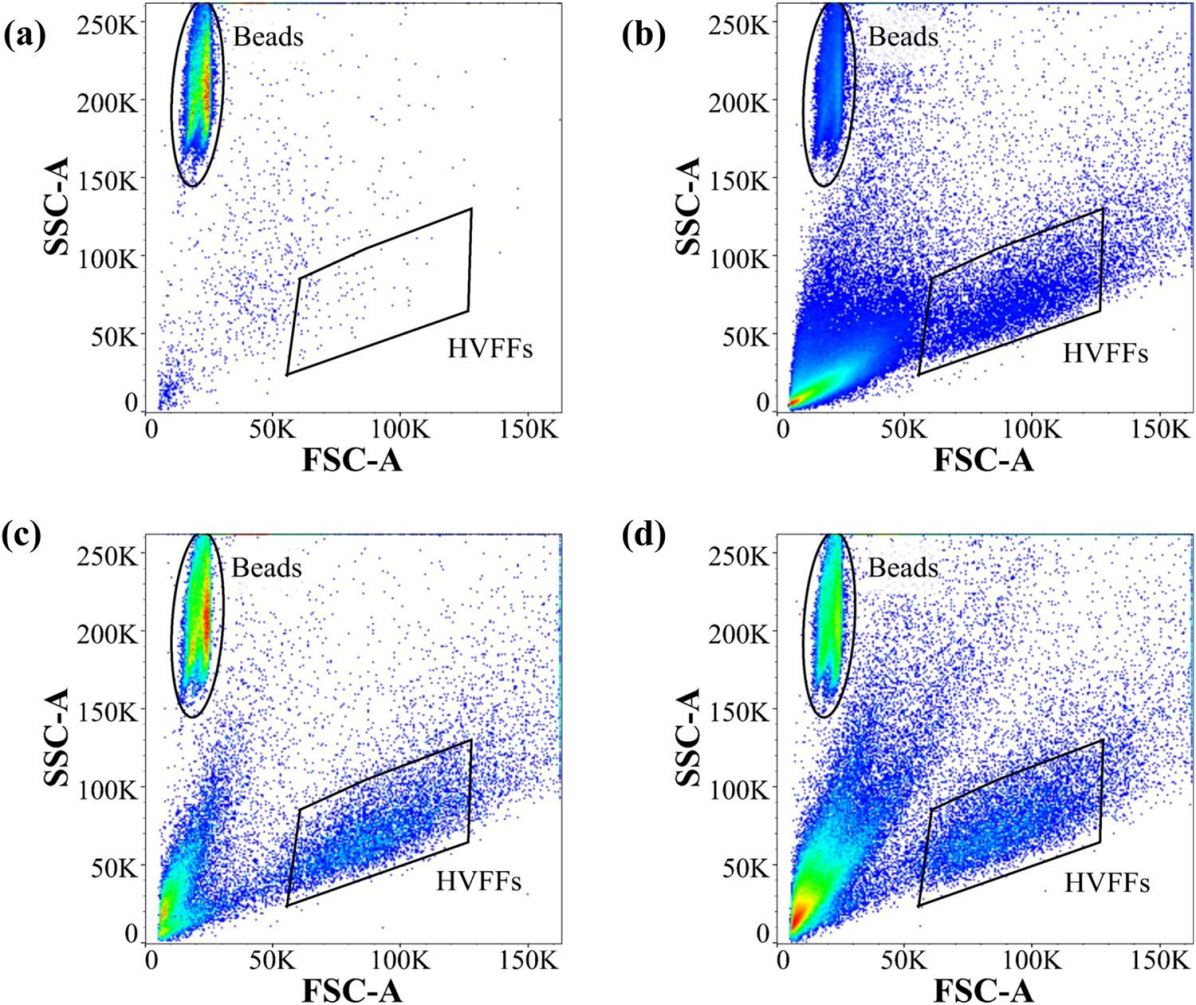
Discrimination of counting beads and migrated fibroblasts based on SSC-A and FSC-A signals in flow cytometric cell counting in the presence of the chemoattractant. A significant increase in the number of migrated cells in CNT composite hydrogels was observed. The events in the left bottom of the graphs are the debris in the cell solution. (**a**) Control, (**b**) CNT250, (**c**) CNT500, and (**d**) CNT750.

Figure 3 shows representative flow cytometric cell counting results for the samples with FBS in the SSC-A vs. FSC-A graphs. Prior to counting the migrated fibroblasts and the beads in FlowJo software, the single particles were isolated using the diagonal polygon gate in forward-scattered height (FSC-H) vs. FSC-A graph. The counting beads used in this study have a high SSC and a low FSC profile. The fibroblasts, however, have lower SSC and higher FSC signals due to their size and intracellular structure. Therefore, the fibroblasts and the counting beads can be clearly discriminated using a polygon and an ellipse gate, respectively, as in Fig. 3. The number of migrated cells for the samples with CNT is significantly greater than that of the control sample.

### Cell adhesion

**Figure 4:**
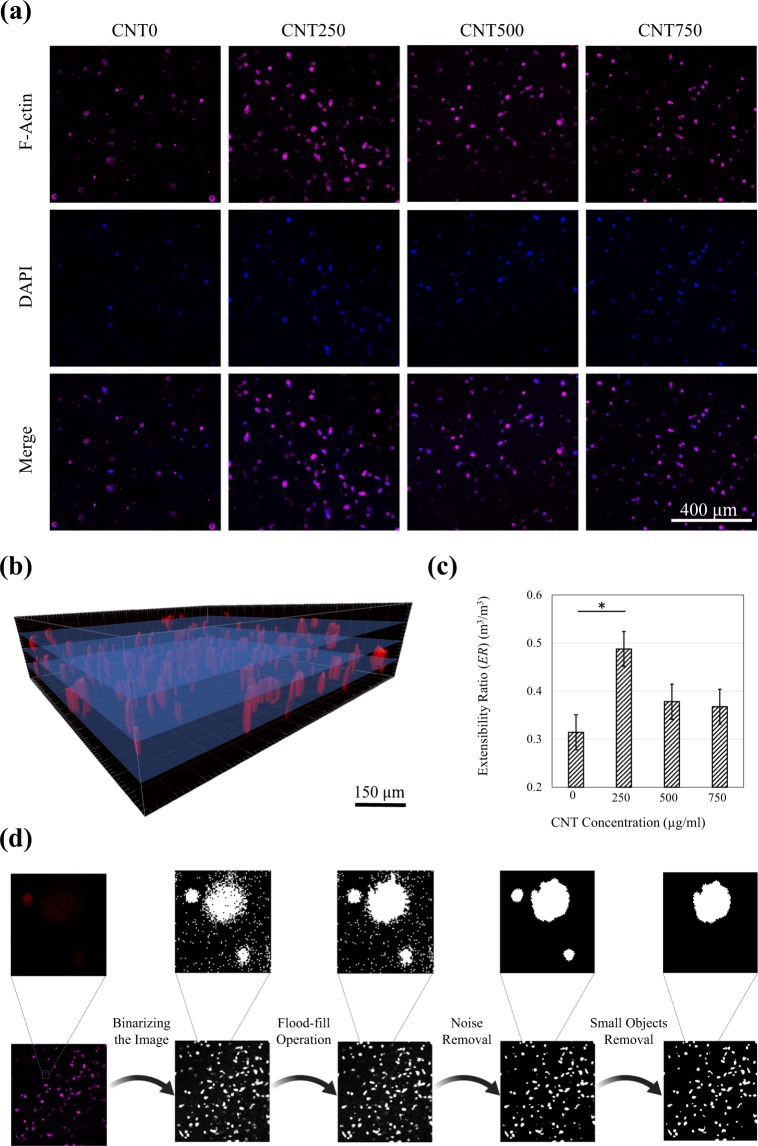
(**a**) Representative confocal images of the F-actin filaments stained with Alexa 633-phalloidin, and the nucleus of the encapsulated fibroblasts stained with DAPI. (**b**) Image segmentation for quantifying cell adhesion in CNT/GC composite hydrogels. (**c**) The extensibility ratio calculated for different groups of the hydrogels acquired from segmented image analysis. *p < 0.05 corresponds to a significant difference. (**d**) Demonstration of the step-by-step image segmentation used to quantify cell morphology and adhesion. The first step was converting the confocal images to binary images. The black pixels which were surrounded by white pixels were changed to white pixels so that a clear and homogenous image of the cell was obtained. After removing noise, the small objects, which consisted of less than 100 pixels, were removed.

The representative images of the F-actin filaments and the nucleus of the encapsulated fibroblasts are shown in Fig. 4a. The 3D confocal laser scanning images of cell adhesion samples were sliced as schematically demonstrated in Fig. 4b, to conduct further morphological analysis. Since the difference in cell morphology is not visually distinguishable, an image processing method was used. The cells morphology was quantified based on the extensibility ratio, *ER*, defined as:

2 $$ER=|1-[(4\pi \frac{S}{{p}^{2}})\times (\frac{L{\prime} }{L{\prime\prime} })]|,$$

where *S* is the surface area of each object in the processed image, *p* stands for the perimeter of the object, and *L*′ and *L*″ represent the length of the minor and the major axis of the ellipse that has the equal normalized second central moments the same as the object, respectively. For a circular object, *i.e*. no adhesion at all, the *ER* value would be equal to 0. When the cells adhere to the hydrogel, the F-actin filaments become elongated, and the objects’ shape changes to a less circular geometry, results in an increase in *ER*. The average value of *ER* for the objects was used to quantify cell adhesion in hydrogels. As shown in Fig. 4c, the CNT250 samples have an average *ER* value higher than the other hydrogels, implying better adhesion. This was not visually detectable in confocal images but became obvious by processing the images and calculating the *ER* for the samples. A representative confocal image of the cells’ F-actin filaments is shown along with a step-by-step processing procedure in Fig. 4d. Each cell constituted one object in the processed image.

### Enzymatic biodegradation

**Figure 5:**
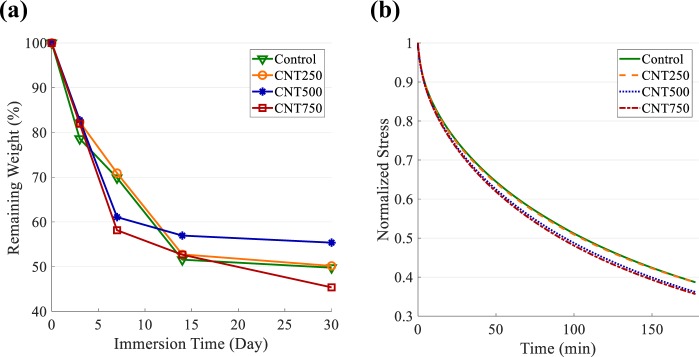
(**a**) The biodegradability of the CNT-based composite hydrogels based on weight loss when immersed in lysozyme solution (Downward-pointing triangle: Control, Circle: CNT250, Asterisk: CNT500, Square: CNT750). (**b**) The decay of shear stress over time for the hydrogels in stress relaxation experiment.

The biodegradation of the CNT/GC composite hydrogels over a time period of one month is shown in Fig. 5a. The samples were degraded to about one-half of their initial weight over this time period. The biodegradation rate during the first week was greater than that over the following weeks. On day 7, the control and the CNT250 samples were degraded to 70% of their initial weight. The two study groups with higher CNT concentration, i.e., CNT500 and CNT750, were degraded to about 60%. Starting from day 14, the degradation rate for all the samples decreased and reached a plateau. In general, the addition of CNTs did not significantly affect the biodegradability of the CNT/GC composite hydrogels.

### Stress relaxation

The time history of the shear stress recorded during the stress relaxation experiment is shown in Fig. 5b. As expected, the stress level decayed over time due to the relaxation of the hydrogel network. The relaxation rate increased for higher concentrations of CNTs. The relaxation timescale (τ^*^) was defined as the time required for the stress level to be relaxed to one-half of its initial value. Based on the definition of τ^*^, the samples with a COOH-CNT concentration of 750 *μg*/*ml* had a 15% lower relaxation time than the control samples, which are CNT-free. The results show that the relaxation time of the GC hydrogel can be slightly modulated by applying different concentrations of COOH-CNT.

## Discussion

The cell recruitment and the 3D chemoattractant-induced cell migration results confirmed that the presence of CNT in GC hydrogel enhances fibroblasts’ migration in the hydrogel network. The average cell migration index is small for all the study groups. This is not specific to CNT/GC hydrogel, as it has been previously reported that in normal *in vitro* conditions only a few percents of fibroblasts migrate through chemotactic mechanisms^40,43^. The cell concentration that we used in the chemoattractant-induced cell migration experiment was less than 7 million cells/ml, which is drastically lower than that of a native vocal fold tissue (66~300 million cells/ml)^44,45^. Higher cell concentration is therefore needed to obtain near-native conditions. The difference between cell migration indices for CNT/GC hydrogels and the pure GC hydrogel, i.e. the control sample, was however statistically significant. The CNT-free GC hydrogel was covalently crosslinked by glyoxal. The addition of carboxylic CNTs resulted in a higher concentration of COOH groups in the hydrogel solution. The abundant COOH groups impeded a complete covalent crosslinking in CNT750 and CNT500. This phenomenon resulted in more floating CNTs within the hydrogels network, which further led to fewer static adhesion sites for the cells in CNT500 and CNT750 samples. However, the CNTs that were firmly positioned in CNT250 hydrogel network were potential focal adhesion sites for the encapsulated fibroblasts. As elaborated in our previous work, the gelation mechanism is faster and more complete in CNT250 samples^17^. The cells, therefore, have more focal adhesion sites for anchoring and migrating. Although the cells cannot fully express their spreading capability in the 3D environment^37^, the trend of changes in the *ER* with respect to the CNT concentration confirmed that the fibroblasts have more affinity to adhere to CNT250 samples. As a result, cell adhesion deems to be a dominant parameter in cell migration rate, as previously has been reported for 2D environments^46^.

Besides cell adhesion and gelation time, other characteristics of the hydrogel may affect the cell migration rate^47,48^. The maximum cell migration occurred in CNT250, while the porosity of CNT750 is the highest^17^. This reveals that although porosity is important for cell recruitment and migration, it does not necessarily play the most important role. Other parameters including, stress relaxation time, and pH can be also influential in cell migration rate within a 3D scaffold. The insignificant difference in biodegradation ratios on day 7 implies that the higher number of migrated cells in CNT hydrogels is not due to faster degradation of the scaffold. This also addresses concerns about the negative effect of CNTs on the biodegradability of GC hydrogel. The degradation of CNT, itself, has been previously studied. It was shown that the CNTs can be enzymatically degraded through shortening and unzipping mechanisms using horseradish peroxidase (HRP)^49^. As a result, the CNT/GC hydrogels may be biodegradable in the human body. However, *in-vivo* experiments should be designed to clearly address the foreign body reaction.

Increasing CNT concentration yielded a lower relaxation time. This can be explained by the fact that the concentration of ionic crosslinks in GC hydrogel is proportional to the COOH-CNT concentration, and the increased ionic crosslinks reduce relaxation time. This trend is in a good agreement with previous studies which compared ionically crosslinked hydrogels with covalently crosslinked hydrogels^50^. Although hydrogels with lower stress relaxation are believed to provide a better substrate for the cells to adhere^39^, cell migration is not necessarily enhanced in hydrogels with faster relaxation. In fact, the addition of COOH-CNT has different effects on cell migration and stress relaxation of the GC hydrogel.

Based on the results of this study and our previous work^17^, two sets of trends were observed for the properties of GC composite hydrogels when carboxylic CNTs were employed. The swelling ratio, porosity, cell viability, and stress relaxation change proportional to the COOH-CNT concentration. The gelation time, storage modulus, electrical capacitance, and cell migration, however, have a local extremum value at the COOH-CNT concentration of 250 *μg*/*ml*.

## Conclusions

We observed that cell recruitment and the cell migration rate significantly increased when COOH-CNTs at concentrations of 250, 500, and 750 *μg*/*ml* were incorporated in a GC hydrogel. The addition of CNTs also changed the morphology of the encapsulated cells and enhanced cell adhesion in the hydrogel. The stress relaxation time changed only slightly with CNT concentration, and the trend of changes was different from that of the cell migration and cell adhesion. Stress relaxation time is therefore not correlated with cell migration. In summary, CNTs could modulate cell migration and adhesion in hydrogels. Enhanced cell migration in injectable implants may accelerate wound healing in different organs of the human body.

## Supplementary information

Cell recruitment in Control sample.

Cell recruitment in CNT250 sample.

Cell recruitment in CNT500 sample.

Cell recruitment in CNT750 sample.

## Data Availability

The raw/processed data required to reproduce these findings cannot be shared at this time as the data also forms part of an ongoing study.
