# Author Correction: Carbon nanotubes promote cell migration in hydrogels

**DOI:** 10.1038/s41598-020-62443-8

**Published:** 2020-03-24

**Authors:** Hossein Ravanbakhsh, Guangyu Bao, Luc Mongeau

**Affiliations:** Department of Mechanical Engineering, McGill University, Montreal, QC H3A0C3 Canada

Correction to: *Scientific Reports* 10.1038/s41598-020-59463-9, published online 13 February 2020

This Article contains errors in Reference 17 which is incorrectly given as:

Ravanbakhsh, H., Bao, G., Latifi, N. & Mongeau, L. G. Functionalized carbon nanotube-based composite hydrogels for vocal fold tissue engineering: Biocompatibility, rheology, and swelling, Materials Science and Engineering: C 109861 (2019).

The correct reference is listed below as Reference 1.
